# Species delimitation based on diagnosis and monophyly, and its importance for advancing mammalian taxonomy

**DOI:** 10.24272/j.issn.2095-8137.2018.037

**Published:** 2018-03-08

**Authors:** Eliécer E. Gutiérrez, Guilherme S. T. Garbino

**Affiliations:** 1 Pós-Graduação em Biodiversidade Animal, Departamento de Ecologia e Evolução, Centro de Ciências Naturais e Exatas, Universidade Federal de Santa Maria, Santa Maria, RS 97105-900, Brazil; 2 Division of Mammals, National Museum of Natural History, Smithsonian Institution, Washington DC 20013-7012, USA; 3 Pós-graduação, Departamento de Zoologia, Instituto de Ciências Biológicas, Universidade Federal de Minas Gerais, Belo Horizonte, Minas Gerais 31270-901, Brazil

**Keywords:** Alpha taxonomy, Phylogenetic Species Concept, Species concepts, Taxonomic inertia, Taxonomic inflation

## Abstract

A recently proposed taxonomic classification of extant ungulates sparked a series of publications that criticize the Phylogenetic Species Concept (PSC) claiming it to be a particularly poor species concept. These opinions reiteratively stated that (1) the two fundamental elements of the "PSC", i.e., monophyly and diagnosability, do not offer objective criteria as to where the line between species should be drawn; and (2) that extirpation of populations can lead to artificial diagnosability and spurious recognitions of species. This sudden eruption of criticism against the PSC is misleading. Problems attributed to the PSC are common to most approaches and concepts that modern systematists employ to establish species boundaries. The controversial taxonomic propositions that sparked criticism against the PSC are indeed highly problematic, not because of the species concept upon which they are based, but because no evidence (whatsoever) has become public to support a substantial portion of the proposed classification. We herein discuss these topics using examples from mammals. Numerous areas of biological research rest upon taxonomic accuracy (including conservation biology and biomedical research); hence, it is necessary to clarify what are (and what are not) the real sources of taxonomic inaccuracy.

## INTRODUCTION

A recently proposed taxonomic classification for extant ungulates (Groves & Grubb, 2011) sparked a series of publications criticizing the species concept upon which the classification was based, i.e., Phylogenetic Species Concept (PSC) (Heller et al., 2013; Zachos et al., 2013; Zachos, 2013, 2015; Zachos & Lovari, 2013), albeit previous published opinions had already presented some of the same arguments against the PSC (e.g., Frankham et al., 2012; Garnett & Christidis, 2007; Isaac et al., 2004; Tattersall, 2007). Two main claims about the PSC have been reiteratively used to highlight it as a particularly poor species concept: (1) the two fundamental elements of the PSC, i.e., monophyly and diagnosability, do not offer objective criteria as to where the line between species should be drawn; and (2) the extirpation of populations can lead to artificial diagnosability and spurious recognitions of species. Moreover, these criticisms portray the use of the PSC as detrimental to conservation efforts. We argue that the problems attributed to the PSC are common to most methodological approaches to species limits and to the most commonly used species concepts that have been the basis for the taxonomic classifications of mammals currently in use. Furthermore, we present evidence that the PSC based on diagnosability and monophyly as operational criteria has helped to substantially advance mammalian systematics. In addition, we show that the recent criticism against Groves & Grubb’s (2011) ungulate taxonomy is mistakenly focused on an "alleged poverty" of the PSC (see a brief comment relevant to this general topic by Tsang et al., 2016, p. 529), whereas the real cause of taxonomic inflation in that proposed classification lays on numerous empirical problems.

## PHYLOGENETIC SPECIES CONCEPT (PSC)

Before we discuss these matters, we must clarify that the name “Phylogenetic Species Concept” has been associated to various concepts (e.g., McKitrick & Zink, 1988; Nixon & Wheeler, 1990), two of which are central in the above-mentioned debate. These concepts can be better regarded as sets of criteria for species delimitation rather than "concepts", as proposed by de Queiroz (2007); however, herein we refer to these sets of criteria as "concepts" only to facilitate communication by using the same terminology employed by authors of previous articles. These concepts are as follows (see summaries by Groves et al., 2017; Zachos, 2016a):

Phylogenetic species concept, diagnosis-based version (dPSC): “*The smallest diagnosable cluster of individual organisms within which there is a parental pattern of ancestry and descent*” (Cracraft, 1983, p.170). Subsequently formulated as “*… the smallest aggregation of populations (sexual) or lineages (asexual) diagnosable by a unique combination of character states in comparable individuals (semaphoronts)*” (Nixon & Wheeler, 1990). A more recent version states that species are “*… the smallest population or aggregation of populations which has fixed heritable differences from other such populations or aggregations*”.

(Groves & Grubb, 2011; see also Groves, 2017)

Phylogenetic species concept, monophyly-based version (mPSC) “*... a geographically constrained group of individuals with some unique apomorphous character, is the unit of evolutionary significance*”.

(Rosen, 1978, p. 176)

A third concept that must be incorporated in the discussion is as follows (see Groves et al., 2017):

Phylogenetic species concept, diagnosis-and-monophyly-based version (dmPSC), defined as “*... the smallest diagnosable cluster of individual organisms forming a monophyletic group within which there is a parental pattern of ancestry and descent*”.

(Mayden, 1997, p. 407; McKitrick & Zink, 1988)

## DO THESE VERSIONS OF THE PSC OFFER OBJECTIVE CRITERIA AS TO WHERE THE LINE BETWEEN SPECIES SHOULD BE DRAWN?

The short answer is “no”, but “no” would also be the answer if the question were asked with regard to any other species concept, including the Biological Species Concept (BSC) when applied to allopatric populations (see Groves, 2012 and references therein). However, the three phylogenetic species concepts described above differ importantly regarding the degree of objectivity with which they can be applied.

With regard to the dPSC (sensu Cracraft, 1983; see also Eldredge & Cracraft, 1980; Nixon & Wheeler, 1990; Wheeler & Meier, 2000 and references therein), we agree with previous criticisms (Heller et al., 2013, 2014; Zachos & Lovari, 2013, Zachos, 2016a) in that this concept is prone to promote spurious recognition of mere geographic (including subspecies) or even individual variants of a single species as if such variants were each a valid species. This is due to the high degree of subjectivity and arbitrariness implicit in the task of judging what characteristics are to be deemed adequate to diagnose species and distinguishing such characteristics from those that would simply lead to diagnoses of populations, or groups thereof, within a single species (but see Wiens & Servedio, 2000). Species are not phenotypically and genotypically homogeneous across geography, therefore it is always the case that various populations within a single species can be diagnosed. These diagnoses by themselves must not be a justification to regard such populations as different species. This limitation of the dPSC is exacerbated when sample sizes are small, as is often the case for medium and large mammals. In these cases, a researcher may erroneously infer the existence of phenotypic discontinuity and the presence of characteristics enabling the diagnosis of a sample—the latter based on a set of specimens that at the time were perceived as worthy of species-level recognition. However, as more samples are obtained, individuals with intermediate phenotypes with respect to the putative new species and other geographic samples may be found. This would render the putative new species, which was previously thought to be diagnosable, conspecific with an already recognized species (e.g., Peres et al., 1996). Examples of these plausible problems are abundant in the proposed classification of ungulates by Groves & Grubb (2011) (see below), but are by no means exclusive to it (e.g., Díaz et al., 1999, 2002; Fonseca & Pinto, 2004; Solari, 2004; van Roosmalen et al., 2000, 2007); numerous examples exist in early contributions to mammalian taxonomy (e.g., Miller, 1912; Pocock, 1941; Robinson & Lyon, 1901), and even the last decade has seen claims advocating for the recognition of a species made on the basis of phenotypic diagnoses of as few as one or two specimens—e.g., Meijaard et al., 2017 p. 513; see also Mantilla-Meluk (2013) for a monkey subspecies named on the basis of morphometric data and pelage coloration from only four specimens. Unfortunately, in some cases descriptions of species have been carried out not only with unacceptably small sample sizes but also merely based on images (illustrations, photos, or both) and lacking preserved type specimens (see Pine & Gutiérrez, 2018 for a review of cases and problems associated to this phenomenon). Although no data exist to support the notion that the collection of a single individual (for it to properly serve as a preserved holotype) significantly increases the probability of an already endangered species to become extinct, some researchers may prefer not to carry out such collection (e.g., Donegan, 2008; but see Dubois & Nemésio, 2007; Dubois, 2009), or it may be unfeasible due to impediments in obtaining collection permits. In such cases, a wide survey of museum specimens might lead to the discovery and subsequent use of specimens in taxonomic descriptions. Undertaking comprehensive surveys of museum specimens may be disregarded by describers of new species, but the possible data yielded, which may be coupled with photos of living animals, might ameliorate the detrimental effects of extremely small sample sizes and help in unveiling geographic and non-geographic (ontogenetic, sexual) variation (e.g., Garbino et al., 2016).

The second concept, the mPSC, under which species must be both monophyletic and geographically restricted, seems indefensible. Within a species there can be large numbers of monophyletic groups that are geographically restricted only due to recent changes in their environment. An example of this is the populations of brocket deer of the Cordillera de Mérida, Venezuela, which for decades were recognized as a valid species, *Mazama bricenii*. This recognition was based on no data whatsoever and on the assumption of a plausible differentiation due to its supposed geographic isolation. However, a recent study that employed ecological niche modeling found that, if these populations were truly isolated in modern time, such isolation commenced not long ago (Gutiérrez et al., 2015). The study also found that while the focal population formed a monophyletic haplogroup, it was embedded within a larger (yet shallow) clade whose terminal branches corresponded to *Mazama rufina*. Results from that study showed that what was once known as *Mazama bricenii* actually corresponds to *Mazama rufina* (Gutiérrez et al., 2015), and illustrate how the application of the mPSC would have led to mistakenly recognize *M. bricenii* as if it were a valid species. Such taxonomic recognition would be a mPSC-based artifact caused by (1) the fact that sequences obtained from specimens from the Cordillera de Mérida (where the populations to which the name *M. bricenii* would apply occur) were recovered in a monophyletic haplogroup; and (2) because those populations might be geographically isolated in modern time.

The third concept, dmPSC, for which species must be both monophyletic and diagnosable, has been useful for improving mammalian taxonomy. As previously noted, many monophyletic groups are found within a single species, and that monophyly per se does not constitute a criterion to determine where the line between species should be drawn. However, it is also true that assessing whether a candidate species is monophyletic or not provides a fundamental basis for its potential recognition as a valid species. Recognizing a polyphyletic taxon as if it were a valid species would be absurd. On the other hand, in some situations (e.g., species originating from peripheral isolation) a candidate species might meet the criteria (i.e., monophyly and diagnosability) for validity under the dmPSC, but its recognition renders the species in which the candidate had thus far been included as paraphyletic. No consensus has been reached as to whether taxonomists should accept paraphyletic species as valid, or, alternatively, if taxonomists should recognize as valid only those that are monophyletic (see Carter et al., 2015; Crisp & Chandler, 1996; Dias et al., 2005; Ebach et al., 2006; Freudenstein, 1998; Funk & Omland, 2003; Hörandl, 2006, 2007; Nelson et al., 2003; Nixon & Wheeler, 1990; de Queiroz & Donoghue, 1988; Zachos, 2014b; Zander, 2007; see also Funk & Omland, 2003). Discussing these views requires a much more extensive text and would distract from the aim of this perspective piece—i.e., clarifying that despite the recent criticisms made against PSCs, at least one of these concepts has served to positively advance mammalian systematics. By requiring monophyly, the application of the dmPSC secures that a phylogenetic inference is conducted to describe or revalidate a species, thus decreasing the chances that polyphyletic groups of populations would be named as a species. These phylogenetic estimates also provide frameworks for evaluating alternatives in cases in which the description or recognition of a clade as a species would render an already recognized species as paraphyletic. In such cases, researchers might simply not describe or formally recognize that clade at all—which might be acceptable, as not every clade in a phylogenetic tree represents a taxon worth naming—or describe it at the subspecies level—with paraphyly persisting at a lower taxonomic rank—or describe it and accept paraphyletic species as valid, in which case the researcher could not invoke any species concept that requires monophyly (including the dmPSC) upon which to base the description. Whichever of these alternatives the researcher prefers, and attempts to justify, due to philosophical, pragmatic, or both considerations, the fact that the dmPSC requires a phylogenetic estimate is an advantage over other concepts that do not, including the dPSC and the BSC.

The requirement that a candidate species must also be diagnosable in order to be recognized under the dmPSC is indispensable. A species must have a series of genetically fixed characteristics that are common to its members and that serve to distinguish it from other such species. However, as already discussed (see above), diagnosability alone is, in general, an inadequate approach to establish species limits.

Acknowledging the existence of the dmPSC is important because it does represent one of the most explicit methods to infer species limits—contra authors that ignored the existence of this concept in their arguments against or in favor of the "PSC" (e.g., Gippoliti et al., 2018; Groves, 2012, 2017; Heller et al., 2013; Zachos & Lovari, 2013; Zachos et al., 2013; Zachos, 2013, 2014a, 2015, 2016a). Some operational steps in delimiting species will always be arbitrary. In this sense, the advantage of the dmPSC over other concepts is that its operational criteria for recognition of species can be objectively tested. In other words, monophyly and diagnosability are, in general, more easily testable for allopatric populations than reproductive barriers (BSC), and more objectively demonstrated than the central criteria upon which other concepts define species, such as “ecological roles” in the Ecological Species Concept (Van Valen, 1976). When applied based on sufficient geographic and taxonomic sampling, and, ideally (but not strictly necessary; see below), employing phylogenetic inferences using data from independent sources (e.g., DNA sequence data obtained from independently inherited genes), the dmPSC has improved the taxonomic classifications of various groups of mammals, some of which remained problematic for decades. Among studies that exemplify how the dmPSC has helped to advance mammalian systematics, even if some of them used this species concept without explicitly or correctly invoking it, are those on didelphid marsupials (e.g., Díaz-Nieto & Voss, 2016; Giarla et al., 2010; Gutiérrez et al., 2010; Martínez-Lanfranco et al., 2014; Pavan et al., 2017; Voss et al., 2018), rodents (e.g., Hawkins et al., 2016; do Prado & Percequillo, 2017; Rogers & González, 2010; Voss et al., 2013), bats (e.g., Baird et al., 2008; Molinari et al., 2017; Moras et al., 2016; Velazco et al., 2010), and medium and large mammals (e.g., Bornholdt et al., 2013; Gutiérrez et al., 2015; Helgen et al., 2009, 2013; Janečka et al., 2008; Koepfli et al., 2008; Miranda et al., 2017; do Nascimento & Feijó, 2017 (and references therein for phylogenetic evidence)). These studies have not only unraveled the true-species nature of previously unrecognized species, but in many cases have shown that taxa considered as valid species for decades are not valid species at all.

The application of any PSC can promote rampant taxonomic inflation when applied without sufficient rigor. In our opinion, this inflation is caused less by philosophical aspects and properties of the PSCs and more by empirical shortcomings. In several studies, geographic and individual variation do not appear to be satisfactorily addressed, and names are applied to what could be intraspecific variants (see examples in Tattersall, 2007). On other occasions, monophyletic groups recovered from molecular phylogenies based on sequence data from a single locus promptly receive new or revalidated names (e.g., Boubli et al., 2012; Thinh et al., 2010). Nevertheless, it is important to note that when the established, traditional taxonomic classification of a focal group is the result of dogmatic acceptance of expert opinions (often past-century authorities), without support from data (see Gutiérrez & Helgen, 2013), then even the use of limited evidence—e.g., analyses of sequence data from a single locus (despite the well-known shortcomings of this approach; see Dávalos & Russell, 2014; Knowles & Carstens, 2007; Maddison, 1997), ideally coupled with qualitative and/or quantitative analyses of morphological data—can well justify taxonomic changes if based on adequate sampling (e.g., Gutiérrez et al., 2010, 2015, 2017; Voss et al., 2013; contra Zachos, 2009, 2016b).

The proposed ungulate taxonomic classification that sparked the recent series of criticism against the PSC is particularly problematic, but not because it was based on the dPSC. Most controversial aspects of this proposed classification are not at all associated to any species concept, as it might seem if one reads the recent debate between Frank Zachos and Colin Groves and their co-authors with regard to the "PSC", but rather to more practical aspects of such a monograph, to name just a few (see also Heller et al., 2013; Holbrook, 2013; Zachos, 2014a, p. 1): (1) Groves & Grubb (2011) did not assess geographic variation at all for most of the species they recognized; (2) unfortunately, for some species recognized by these authors, the sample size employed was not indicated nor any published study cited to support the taxonomic proposals, whereas for many other alleged species the sample sizes were extremely low—e.g., *Alcelaphus tora*, *Dorcatragus megalotis*, *Eudorcas nasalis*, *Eudorcas tilonura*, *Gazella acaciae*, *Gazella karamii*, *Gazella shikarii*, *Lama mensalis*, *Madoqua hararensis*, *Mazama fuscata*, *Mazama jucunda*, *Mazama trinitatis*, and *Redunca cottoni*; (3) no published phylogenetic information seems to be the basis of most of their taxonomic propositions; (4) in general, no detailed discussions were presented on whether recognizing a taxon as a valid species was more appropriate and justifiable than regarding it as a subspecies, and it seems that the objective of the authors was to merely recognize as valid species as many taxa as possible, without critical evaluation of alternatives (e.g., recognizing subspecies when appropriate); (5) a list of the specimens examined was not provided, and hence it is difficult for the scientific community to evaluate the authors’ assertions on specimen morphologies based on the same material with certainty; (6) no data were made available that would enable reproduction and testability of the analyses that were the basis of the taxonomic propositions; (7) although the authors cite published studies for some of the taxonomic changes they proposed, for others they did not and nowhere in their monograph can be found results from any quantitative analyses. We cannot understand why Groves & Grubb (2011) failed to publish the results of their quantitative analyses given current possibilities to do so (see below). Unfortunately, this problem is not unique to the proposed ungulate taxonomic classification of Groves & Grubb. An important volume on mammals of South America (Patton et al., 2015) contained the first modern taxonomic treatments of various rodent groups (e.g., family Sciuridae, genera *Aepeomys*, *Oecomys*, *Rhipidomys*, *Thomasomys*), but results from analytical procedures assessing geographic and non-geographic variation of those groups have not been published (in the book or elsewhere), and in some cases it seems unlikely they will ever be published. Luckily, several unpublished Ph.D. dissertations that served as the basis for the book sections treating those taxa have been privately shared among colleagues. Clearly, making these Ph.D. dissertations (and other unpublished material) digitally available to the scientific community free of charge from a repository on the Internet (e.g., Dryad, Figshare, Internet Archive, ResearchGate, Zenodo), if their authors grant authorization, should be considered by the editors of this book, and similar actions should be considered by authors and editors of future monographs introducing taxonomic classifications.

## CAN EXTIRPATION OF POPULATIONS LEAD TO ARTIFICIAL DIAGNOSABILITY AND SPURIOUS RECOGNITIONS OF SPECIES UNDER THE dmPSC?

**Figure 1:**
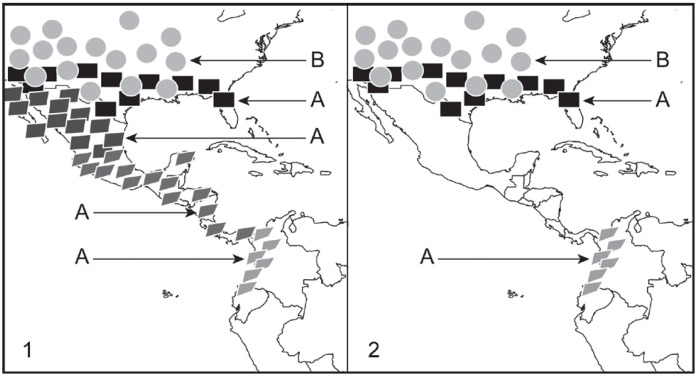
Illustration of how population extirpations can promote artifactual recognition of populations of a single species as if they were different species under the Biological Species Concept (and many other concepts)

The short answer is "it can", but again the same answer would apply if the question were asked about other species concepts, including the BSC. In their criticism of the "PSC", Zachos & Lovari (2013) claimed that the fact that extirpation of populations can lead to artifactual diagnosability and monophyly is one of the weaknesses of the PSC that makes this concept a particularly poor one. They stated that “*There is yet another line of argumentation that clearly shows the shortcomings of both diagnosability and monophyly as yardsticks for species delimitation and that we believe is another coup de grâce for the PSC. Diagnosability (just like reciprocal monophyly) can and often does occur as a consequence of extinction of intermediate forms...*”. Unfortunately, Zachos & Lovari (2013) did not realize that extirpation of intermediate populations is one of the natural causes of speciation. These events lead to speciation, affecting gene flow and ecological adaptation of extreme phenotypes in populations that previously were genetically connected by the existence of intermediate populations. In fact, it could be said that living taxa that can be validly recognized on the planet exist as separate biological entities and as taxonomically diagnosable units only because of the extinction of intermediate forms. If all intermediate forms that have lived since the beginning of life on Earth were still with us, then all living organisms, from prokaryotes to eukaryotes and from plants to animals, would exist as a single morphological and reproductive continuum: no distinguishable taxa would exist! Logically, in cases in which extirpations have taken place fairly recently (e.g., due to human-related causes) no speciation may have yet occurred. In such cases, the extirpation of populations can potentially lead to artifactual recognitions of species under the PSCs, but this issue is far from being associated only to the PSCs; rather, it is a problem that can potentially affect most, if not all, species concepts currently in use. For example, this issue can lead to artifactual recognition of species under the BSC. Let us imagine a species, species A, with wide distribution and showing geographic variation by way of a cline in several qualitative cranial traits believed to be taxonomically important, i.e., used by most authors to distinguish species within the corresponding genus. We will illustrate these traits as color and shape in polygons that represent populations of species A (Figure 1, panel 1). If recent extirpations of intermediate populations take place and only those populations occurring at the opposite extremes of species A’s range remain as extant, then these populations will become allopatric and it would be highly likely that they would be considered as members of different species under the BSC (Figure 1, panel 2). The BSC would fail to regard these populations as conspecific, even employing the approach presented by Tobias et al. (2010; see also Brooks & Helgen, 2010), which uses the degree of differentiation known to exist between different but sympatric species (i.e., species A and B in Figure 1) as a standard to assess the taxonomic relevance of differentiation between allopatric populations (i.e., populations of species A in North and South America in Figure 1)—in order words, this approach uses the former degree of differentiation as a threshold at which (or above) allopatric populations could be treated as different species under the BSC.

Species A, which is represented by quadrilaterals (rectangle and rectangle-like polygons), possesses a wide distribution and geographic variation by way of a cline in several qualitative cranial traits considered taxonomically important; herein these traits are illustrated as color and shape of the polygons (panel 1). Species B, which is represented by circles, is restricted to North America, where it occurs in sympatry with some North American populations of species A (panel 1). If extirpation of populations of species A takes place and only those populations occurring at the opposite extremes of species A’s range remain as extant, then these populations will become allopatric (panel 2). In that scenario, it would be highly likely that the remaining extant populations would be considered as members of a different species under the BSC (panel 2). The BSC would fail to regard these populations as conspecific, even employing the approach presented by Tobias et al. (2010; see also Brooks & Helgen, 2010), which uses the degree of differentiation known to exist between different but sympatric species (i.e., species A and B) as a standard to assess the taxonomic relevance of differentiation between allopatric populations (i.e., populations of species A in North and South America).

## CONCLUSIONS

Major differences exist among the different concepts labeled as the "Phylogenetic Species Concept", and the one that uses both diagnosis and monophyly (dmPSC) to delimit species has been, and will continue to be, important for positively advancing mammalian taxonomy. Our preceding discussion should rectify misunderstandings that could arise from claims made in recently published opinions debating alleged pros and cons of the "PSC". Although we partially agree with some of the arguments presented by participants of that debate, the proposed taxonomic classification of ungulates (Groves & Grubb, 2011) that motivated this debate is highly deficient, in our view, not so much because of the species concept it employed (i.e., dPSC), but rather due to serious empirical problems. Among them is the absence of statistical assessments of geographic and non-geographic variation in diagnostic traits. Although in many instances the number of specimens available in museums should have permitted statistically satisfactory assessments of geographic and non-geographic variation, results from those analyses were not presented by Groves & Grubb (2011) in their monograph. In other instances, extremely low sample sizes precluded proper statistical analyses.

To refrain from producing taxonomic hypotheses because of limited material (e.g., few available museum specimens) would hamper progress in medium and large mammal taxonomy. As previously mentioned, collecting new samples of such mammals can be logistically impracticable, and some researchers may simply prefer not to collect them due to conservation concerns (but see clarification above). Thus, even when the available material consists of only a few specimens, taxonomic studies should still be carried out, but the taxonomist should bear in mind the statistical limitations of a small sample, such as inadequate estimations of population ranges and lower confidence levels. Collating information from multiple data sources, such as nucleotide sequences, discrete and continuous morphological data, and behavior—an approach nowadays called “integrative taxonomy” (e.g., Dayrat, 2005)—has long been considered useful as it theoretically increases the probability of correctly identifying and delimiting taxonomic entities (Simpson, 1961; Tinbergen, 1959).

Numerous areas of biological research rest upon taxonomic accuracy (including conservation biology and biomedical research); hence, it is necessary to clarify what are (and what are not) the real sources of taxonomic inaccuracy.

## Supplementary Material

http://www.zoores.ac.cn/fileup/2095-8137/SUPPL/20180720191247.zip
